# Kikuchi-Fujimoto Disease: A Rare Cause of Pyrexia of Unknown Origin and Cervical Lymphadenopathy

**DOI:** 10.7759/cureus.30823

**Published:** 2022-10-29

**Authors:** Abdalla Fadul, Eihab A Subahi, Elrazi A Ali, Habeeb Awadalkareem, Gihan Mohamed, Mohamed Elawad, Mohammad Sharaf Eldean, Adel Albozom

**Affiliations:** 1 Internal Medicine, Hamad Medical Corporation, Doha, QAT; 2 Internal Medicine, One Brooklyn Health/Interfaith Medical Center, Brooklyn, USA; 3 Pediatrics, Hamad Medical Corporation, Doha, QAT; 4 Radiology, Hamad General Hospital, Doha, QAT; 5 Histopathology, Hamad Medical Corporation, Doha, QAT

**Keywords:** kikuchi-fujimoto disease, puo (pyrexia of unknown origin), unexplained fever, necrotizing granulomatous lymphadenitis, cervical lymphadenopathy

## Abstract

Kikuchi-Fujimoto disease (KFD) is a benign disorder characterized by regional cervical lymphadenopathy with tenderness. Associated symptoms of KFD include low-grade fever, night sweats, weight loss, nausea, and sore throat. The disease is a sporadic disease known to have a worldwide distribution with a higher prevalence among Asian communities. Although the clinical and histopathological features point to a viral etiology, this hypothesis has not been proven yet. Generally, the diagnosis is made based on a lymph node excisional biopsy. Its recognition is crucial mainly because this disease can be mistaken for other disorders, including systemic lupus erythematosus or malignant lymphoma. Supportive treatment includes antipyretics, non-steroidal anti-inflammatory drugs, and corticosteroids. Spontaneous recovery occurs within a few weeks. Patients should be followed up for years to survey because there is a possibility of developing systemic lupus erythematosus.

In this article, we report the case of a patient who presented with a fever of unknown origin and lymphadenopathy, treated with multiple antibiotic courses with no improvement. Workup including computed tomography of the neck with contrast and lymph node biopsy confirmed the diagnosis of KFD. His condition improved after administering analgesics and multivitamins, and he was advised to rest at home.

## Introduction

Kikuchi-Fujimoto disease (KFD), also known as histiocytic necrotizing lymphadenitis, was initially reported separately in 1972 by Kikuchi and Fujimoto. The early manifestations of KFD are cervical lymphadenopathy, fever, headache, and tiredness. The cause of the disease is unidentified, although viruses and autoimmune mechanisms have been suggested [1].

Most cases are reported in Asia, despite cases reported globally in the literature. The occurrence of KFD is elevated in females in the age group of 20-35 years, with a female-to-male ratio of incidence of 4:1 [2].

It is crucial to identify KFD as it may resemble other differential diagnoses such as lymphoma, infectious causes, and autoimmune illnesses such as systemic lupus erythematosus. As reported by one study, 30% of cases of KFD are incorrectly diagnosed as lymphoma [3].

Laboratory results of KFD may show low white blood cell count, low hemoglobin, high erythrocyte sedimentation rate, and high C-reactive protein. Diagnosis of KFD is made on biopsy and histopathologic examination and ruling out other differentials. The disease is self-limited. Treatment includes supportive measures, and the manifestations resolve spontaneously within four months most of the time [4].

## Case presentation

A 46-year-old Indian gentleman with no significant medical history presented with a fever for one month. The patient had been on vacation in India and came back to Qatar four weeks prior to admission. His fever started while he was in India. The fever was mild initially and then started to worsen. He took many antibiotics with no improvement, including cefixime, azithromycin, augmentin, and ceftriaxone.

One week before the presentation, he noticed a swelling on the left side of the neck (supraclavicular region). The swelling was tender and associated with fever without any specific timing, resolved with paracetamol, and no night sweats. No other family members were sick, and he had no personal or family history of tuberculosis or malignancy. Although he was in contact with a coronavirus disease 2019 (COVID-19)-positive patient in India, his COVID-19 antigen test was negative. He also reported a loss of appetite with a 2 kg weight loss in the previous month. His social history was positive for smoking one to two cigarettes per day and drinking alcohol regularly for about 10 years. His immunizations were up to date, and he had received a three-dose COVID-19 Moderna vaccine.

**Figure 1:**
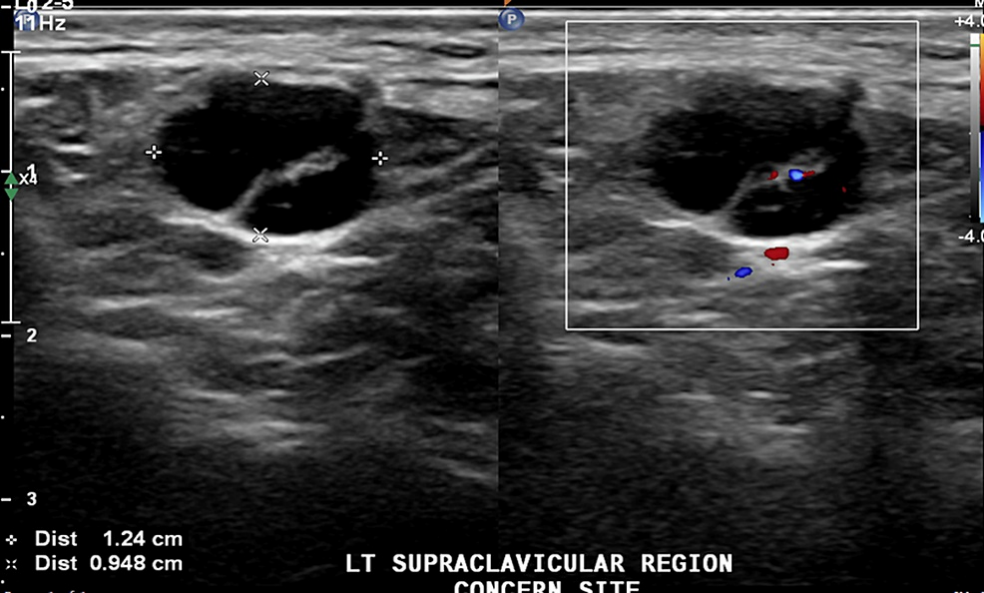
Grayscale (left) and color Doppler (right) ultrasound of the left supraclavicular region shows enlarged round lymph nodes with preserved fatty hilum and normal vascularity, measuring 9.5 mm in the short axis.

**Figure 2:**
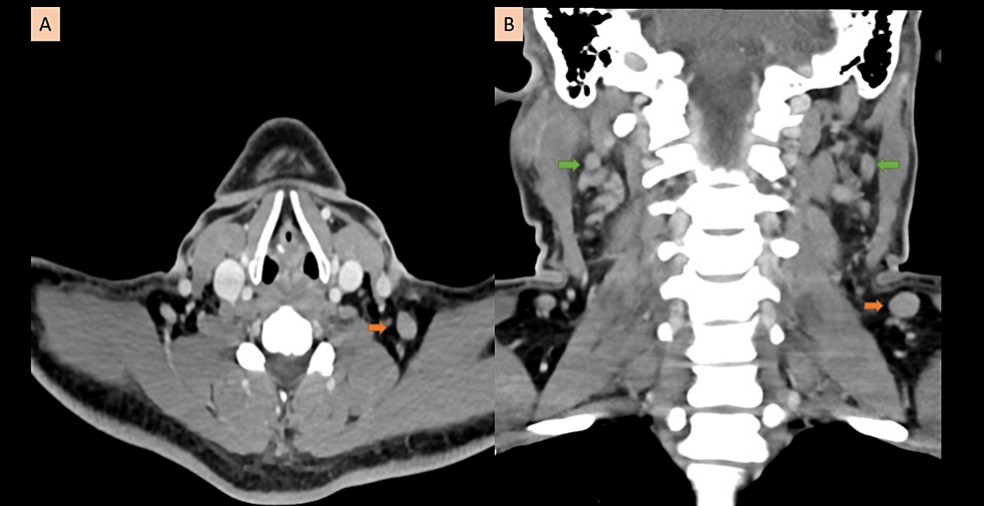
Axial (A) and coronal (B) computed tomography of the neck with contrast show left supraclavicular enlarged lymph nodes but no necrosis or calcification (orange arrow). Multiple bilateral reactive deep cervical lymph nodes can be seen (green arrow).

On examination, he had enlarged left supraclavicular nodes, tender and measuring around 1 × 1 cm. Ultrasound of the neck showed multiple lymph nodes in the left supraclavicular region, and multiple jugular lymph nodes were noted bilaterally. Computed tomography (CT) confirmed a suspicious left supraclavicular lymph node. Differential diagnoses included lymphoma/lymphoproliferative disorders and inflammatory causes (Figures 1, 2).

**Figure 3:**
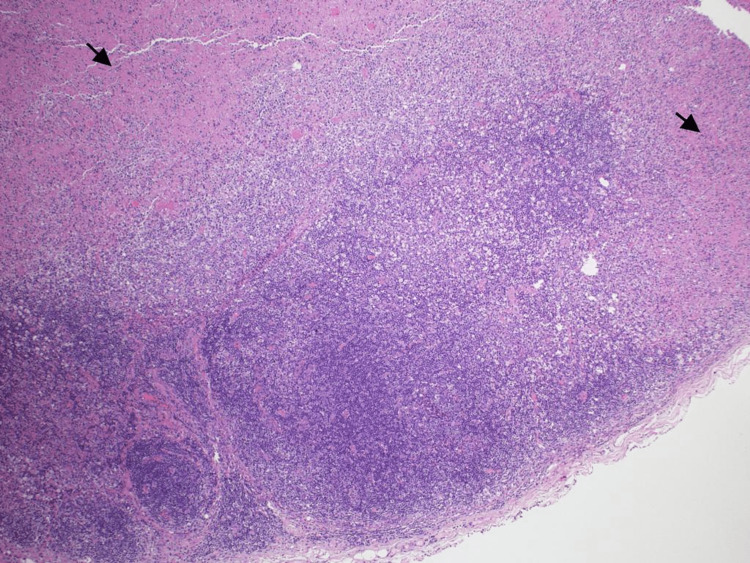
Light microscopic examination showing a lymph node with patchy necrotic areas (arrowheads) (hematoxylin and eosin, ×40).

**Figure 4:**
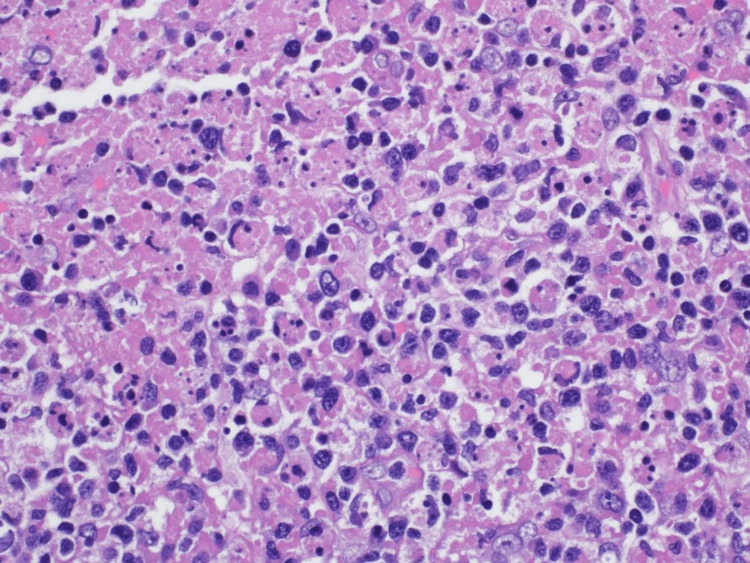
Light microscopic examination showing the eosinophilic necrotic area with karyorrhectic debris on the left side surrounded by sheets of plasmacytoid histiocytes and lymphocytes on the right side (hematoxylin and eosin, ×400).

A biopsy of the left supraclavicular lymph node was done. Light microscopic examination revealed a distorted lymph node with patchy necrotic areas showing a moth-eaten appearance. These necrotic areas consisted of amorphous eosinophilic material admixed with karyorrhectic debris (Figures 3, 4).

At the periphery of these necrotic areas were sheets of plasmacytoid histiocytes mixed with foamy macrophages, lymphocytes, and immunoblasts. Special stains for acid-fast organisms (Ziehl-Neelsen stain) and fungi (Grocott methenamine silver) were negative. Immunohistochemistry for viral inclusions of cytomegalovirus (CMV), herpes simplex virus (HSV)-1, and HSV-2 was negative. In situ hybridization for Epstein-Barr virus (EBV) was negative. Flow cytometric examination failed to demonstrate any monoclonal proliferation of B lymphocytes or T-cell receptor rearrangement of T lymphocytes. The patient was given analgesics and multivitamins and was advised to rest at home. His condition improved and he was asked to follow up in the clinic.

## Discussion

Benign histiocytic necrotizing lymphadenitis is also known as KFD. Drs. Kikuchi and Fujimoto separately discovered the unusual syndrome in 1972. They presented a case of lymphadenitis marked by a localized proliferation of reticular cells, several histiocytes, and significant nuclear debris [5]. A few cases have been documented in America, Europe, and Africa of this disease, which often affects women in their third to fourth decades of life, and is especially prevalent among those of Asian descent [6]. AlSoub et al. reported a male-to-female ratio of 2.5:1 in Qatar, in contrast to other reports that indicated a female predominance, with ratios ranging from 4:1 to 6:1 [7].

KFD is thought to either occur as a response to a viral infection or due to an underlying autoimmune disorder [7]. The discovery of histiocytes and CD8-positive cells in KFD-affected lymph nodes supports the viral origin. Numerous studies have attempted to show an association between KFD and different viruses. In a study by Cho et al., polymerase chain reaction (PCR) was used to check 50% of lymph node tissues identified with KFD for the presence of human herpesvirus (HHV-6), 7, and 8. This study could not establish a link between KFD and HHV-6, 7, or 8 [8]. Hudnall et al. examined 30 lymph nodes affected by KFD and demonstrated that HHV-1, varicella-zoster virus, and HHV-8 DNA were not detectable, and HHV-2, CMV, HHV-6, and HHV-7 were occasionally detected [9]. This contrasts with another study by Zhang et al. which identified an association between parvovirus B19 and KFD. According to the study by Hudnall et al., it was unlikely that these viruses served as the etiology of KFD [10].

It has also been demonstrated that autoimmune disorders likely play a role in the pathogenesis of KFD. A study by Sopeña et al. detected autoimmune conditions, including SLE, thyroiditis, leukocytoclastic vasculitis, Sjogren’s syndrome, Still’s disease, and Wegener’s granulomatosis, which was also reported in another study [11]. Kucukardali et al. reported 32 cases of KFD associated with SLE. Of these instances, 18 had KFD and SLE at the same time, six developed SLE later, and four had a pre-existing condition that was indicative of SLE [12]. Goldblatt discussed three Asian women who had KFD. None of the three patients had symptoms or signs typical of SLE at the time of their diagnosis, and testing for antinuclear antibodies (ANA) was negative for all three patients. However, after receiving a diagnosis of KFD, all three patients experienced symptoms of SLE and a positive ANA test within a 3-14-month window. Although the link between KFD and SLE has been reported, the exact association remains unclear [13].

Based on the clinical presentation, the most extensive published series of 244 KFD cases revealed that lymphadenopathy (100%), erythematous rashes (10%), and arthritis (7%), in addition to fever (35%), are the most common symptoms. The following clinical characteristics are also frequently reported: rash (10%), fatigue (7%), and hepatosplenomegaly (3%). In individuals with extranodal illness, less common symptoms such as weight loss, night sweats, sore throat, nausea, and vomiting have been more reliably recorded. Skin lesions such as papules, plaques, nodules, and facial malar erythema are the most prevalent presentation in those with extranodal illness and may be present in up to 40% of the patients [6]. Some incidences of aseptic meningitis have also been documented. Although cases of 5-6 cm lymph nodes have been reported, the typical lymphadenopathy linked to KFD is 3 cm. According to the literature, fever can last anywhere between one and seven weeks, with temperatures ranging between 38.6°C and 40.5°C [14]. Although leukopenia has been observed to occur in up to 50% of cases, patients with KFD often have normal blood counts. Peripheral blood films have also revealed atypical lymphocytes in 25% of patients. Other laboratory results include increased serum erythrocyte sedimentation rate, lactate dehydrogenase, and mildly abnormal liver function tests [15].

It is uncommon for imaging techniques such as CT, magnetic resonance imaging, and positron emission tomography/computed tomography scanning to produce definitive diagnostic results. Using imaging alone to make a preliminary diagnosis of tuberculosis or lymphoma is frequently inaccurate. In a study of 96 retrospective scans of confirmed KFD cases, Kwon et al. found that KFD cases had the following characteristic appearances on CT scanning: it is possible to distinguish numerous homogeneous lymphadenopathy, including levels II to V from lymphoma because 94% of the nodes were smaller than 2.5 cm, whereas lymphoma often produces a few but larger nodes. Perinodal infiltration and necrosis are also frequently observed [16].

Frequently, lymph nodes with a hypoechoic center and a hyperechoic rim can be seen on ultrasound imaging. Again, the specificity of these criteria for KFD is minimal. These results rule out using imaging techniques to diagnose KFD. However, linked to the clinical history, one may at least be suspicious of KFD rather than a neoplastic lesion [17]. A tissue biopsy or a whole lymph node biopsy provides a definitive diagnosis. Characteristic findings are seen after histopathological evaluation of the afflicted lymph nodes. Proliferative, necrotizing, and xanthomatous patterns are the three main types found. A dominating inflammatory infiltrates present in the proliferative image, which is present in about one-third of patients. A necrotizing pattern is present in 50% of cases, while the xanthomatous type is uncommon and has many foam cells [18]. The differential diagnosis of KFD includes lymphoma, tuberculosis, SLE, blastic plasmacytoid dendritic cell neoplasm, Kawasaki’s disease, sarcoidosis, HSV, EBV, and metastatic adenocarcinoma [19].

KFD is often considered a mild, self-limiting illness that cures within six months. Relapses are reportedly possible in 3-4% of patients. Analgesia, antipyretics, and non-steroidal anti-inflammatory drug therapy are crucial treatment components after a diagnosis. Although uncommon, corticosteroid treatment is advised if extracervical or extranodal illness is present [20]. Individuals may require surveillance for SLE after recovering from KFD due to its probable link with SLE [21].

## Conclusions

The rarity and variable clinical presentation of KFD create significant diagnostic and therapeutic challenges for clinicians. This article aims to increase awareness of this entity’s differential diagnosis of pyrexia of unknown origin and cervical lymphadenopathy. Because pyrexia of unknown origin with lymphadenopathy is a joint presentation for patients in internal medicine and emergency department settings, KFD is likely to be underdiagnosed and underrecognized in this population. With a wide range of possible diagnoses, such as infection, cancer, SLE, and tuberculosis, knowing the possibility of KFD may help avoid or delay invasive tests such as bone marrow biopsies. Family members should also feel better knowing that the ailment is self-limiting for the patient.

One to four months after a diagnosis, the cervical lymphadenopathy resolves independently. Studies should continue to learn more about KFD due to its diverse clinical presentation and unusual rarity. The hunt for the disease’s primary etiology should never stop. Clinical research has revealed that KFD might develop due to an immunological reaction to underlying viral infections, bacterial infections including tuberculosis, autoimmune disorders, or viral diseases. Therefore, it is necessary to investigate the disorders linked to KFD to understand how these illnesses are related. Even though KFD is a self-limiting condition, individuals may require surveillance for SLE after recovering from KFD due to its probable link with SLE.
